# Supplementary material for: MetaRibo-Seq measures translation in microbiomes
Source: Nat Commun. 2020 Jun 29;11:3268. doi: 10.1038/s41467-020-17081-z (PMC7324362; doi:10.1038/s41467-020-17081-z)
Supplement: Supplementary file 10 — Supplementary Data 7 [file 41467_2020_17081_MOESM10_ESM.zip › File2/Confidence_High_Taxonomy/252741_out.krona.html]

Javascript must be enabled to view this page.

members
magnitude
magnitudeUnassigned
count
unassigned
taxon
rank

252741\_out

5

2
superkingdom
5

phylum
976
5

class
200643
5

5
order
171549

171552
family
5

838
genus
5

5

SRS015782\_contig\_number\_contig-100\_429.250070SRS047044\_contig\_number\_14306SRS097958\_contig\_number\_11377SRS144297\_contig\_number\_9290SRS144714\_contig\_number\_24370
165179
species
